# Temporary Perineal Urethrostomy for Bladder Tumor Resection

**DOI:** 10.1155/criu/1639039

**Published:** 2026-08-27

**Authors:** Avaneesh Kunta, Audra Garrigan, Kevin Heinsimer

**Affiliations:** ^1^ University of Central Florida College of Medicine, Orlando, Florida, USA, ucf.edu; ^2^ Department of Urology, USF Health Morsani College of Medicine, Tampa, Florida, USA, usf.edu

**Keywords:** bladder cancer, burn scarring, case report, perineal urethrostomy, TURBT

## Abstract

We report a case of a 57‐year‐old male with extensive pelvic scarring from childhood burns who presented with gross hematuria and was diagnosed with high‐grade pT1 urothelial carcinoma. Due to severe anatomical restrictions, traditional transurethral resection of bladder tumors (TURBT) was not feasible. A temporary perineal urethrostomy (PU) was performed to enable complete tumor resection. This is the first reported case of temporary PU facilitating TURBT in a patient with severe burn‐related pelvic scarring, highlighting its role as a safe and effective alternative when standard endoscopic approaches are not possible.

## 1. Introduction

Perineal urethrostomy (PU) is a lesser‐utilized surgical approach for accessing the bladder in patients who have anatomical difficulties that make conventional transurethral resection of bladder tumors (TURBT) challenging. Patients with severe urethral stricture disease, pelvic trauma, and conditions that limit the mobility of the penis or surrounding tissues may hinder traditional endoscopic access [1, 2]. PU provides direct access to the bladder and lower urinary tract through a midline perineal incision, allowing the urologist to bypass the urethra when it is obstructed or damaged, as well as ensuring the safe resection of bladder tumors [2, 3].

Temporary PU has shown success in patients undergoing complex surgeries for benign prostatic hyperplasia (BPH) and bladder tumors, when standard rigid instruments cannot traverse the urethra due to anatomy [2]. In this report, we present the case of a 57‐year‐old male with extensive pelvic scarring from childhood burns, which necessitated the use of a temporary PU to enable complete transurethral resection of high‐grade bladder tumors.

## 2. Case Presentation

A 57‐year‐old male with a history of 70% body surface area burns as a child presented to an outside hospital with complaints of difficulty urinating and gross hematuria for 6 weeks. On cystoscopy, he was found to have a bladder tumor. He went to the operating room; however, a rigid cystoscope was unable to be passed due to severe limited mobility of the patient′s tethered skin and therefore a flexible cystoscopy was performed. Cystoscopy demonstrated a large, papillary tumor with biopsy of high‐grade, invasive urothelial carcinoma pT1. The tumor was unable to be completely removed therefore he was referred to our institution for further management.

**Figure 1:**
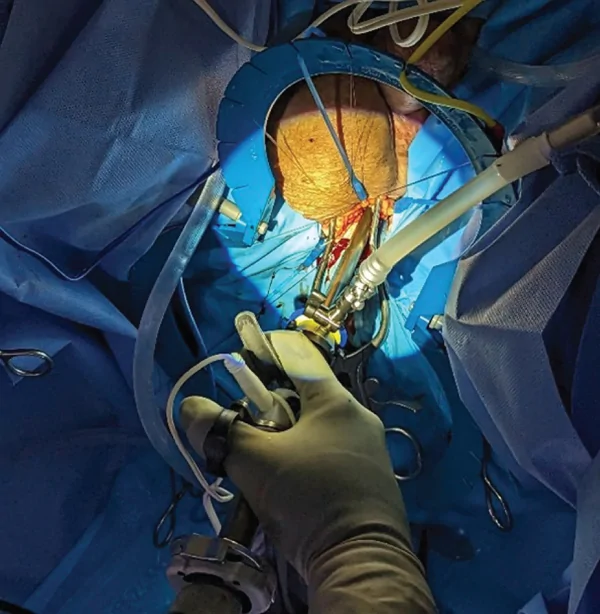
Resectoscope via perineal urethrostomy.

After confirming the presence of tumor on clinic cystoscopy, the patient was taken to the OR shortly thereafter. An initial attempt was made to perform cystoscopy with a 22 Fr rigid cystoscope. However, the patient′s pelvic scar tissue and limited mobility of the lower abdominal skin prevented successful entry into the posterior urethra, making a standard transurethral approach impossible. This was consistent with the referring urologist′s experience. The anterior urethra was normal, but the inability to navigate through the scarred tissue led to the decision to perform a TURBT via a temporary PU (Figure 1). Given his known history, he had been consented for this preoperatively.

A 3‐cm midline perineal incision was made along the median raphe, carried down through the subcutaneous tissue to expose the bulbocavernosus musculature. The bulbocavernosus muscle was divided along the midline to expose the underlying bulb of the urethra. A urethral sound was introduced into the urethra to confirm its location. Next, the urethra was incised ventrally for 1.5 cm in the proximal bulbar urethra so the scope would enter the urethrostomy parallel to the floor. The 26 Fr resectoscope was then successfully inserted and advanced into the bladder. TURBT revealed three large frondular bladder tumors at left posterior bladder wall, left dome, and right bladder neck. The tumors were able to be completely resected. Hemostasis was achieved. The urethrostomy was closed using interrupted 4‐0 Monocryl for the urethra and running 3‐0 Monocryl for the bulbospongiosus muscle and Colles fascia. Skin was closed with a running 4‐0 Monocryl. A 20 Fr two‐way Foley catheter was placed through the meatus without difficulty and left in place for 2 weeks.

At his 2‐week postoperative appointment, the patient was found to have developed superficial wound dehiscence at the perineal incision site, requiring a return to the operating room for wound washout and closure. There was no evidence of urethral leakage or fistula. The patient recovered without further complications and the Foley was removed 1 day after wound closure. The patient′s final pathology was high‐grade noninvasive Ta urothelial carcinoma with muscle in the specimen without tumor invasion.

Three months postoperatively, the patient returned to our institution and was found to have recurrent bladder tumors. He had been arranged to complete intravesical Bacillus Calmette–Guérin (BCG); however, he was unable to complete treatment due to transportation and scheduling barriers that limited his ability to attend weekly clinic visits. Given the small size of the tumors, they were treated via flexible cystoscopy with biopsy and standard electrocautery fulguration. Pathology confirmed recurrent high‐grade Ta disease. Because the recurrent tumors were small and located in areas accessible with flexible cystoscopy, a temporary PU was not required for subsequent management. Now 15 months out from his first temporary PU, he continues to undergo management and close surveillance for his bladder cancer but has had no urethral strictures or other complications from his PU. The urethra was primarily realigned at the time of initial PU closure, and no evidence of stricture has been identified on subsequent surveillance cystoscopy; accordingly, no urethroplasty, suprapubic realignment, or intermittent self‐catheterization has been required.

## 3. Discussion

This case is the first of its kind to illustrate the successful use of a temporary PU to address the challenge of bladder tumor resection in a patient with severe pelvic scarring due to childhood burns. Traditional TURBT was impossible due to limited mobility of the patient′s lower abdominal skin, which restricted the use of rigid cystoscopic equipment. Temporary PU provided an effective and safe alternative to access the bladder, allowing complete resection of the tumors without significant complications. Interestingly, the initial biopsy demonstrated high‐grade pT1 disease, whereas the final resection pathology demonstrated high‐grade pTa with muscularis propria present and uninvolved. This discrepancy may be explained by sampling error in the initial biopsy and tumor heterogeneity, in which small invasive foci may coexist with predominantly noninvasive papillary disease. It is important to emphasize that the indication for PU in this case was anatomical, reflecting the inability to pass a rigid cystoscope through severely scarred, immobile tissue, rather than an a priori assumption of invasive disease. Complete resection with adequate detrusor muscle sampling was necessary regardless of the eventual stage, since accurate depth of invasion cannot be reliably determined from a partial or endoscopically limited biopsy, and the outside biopsy had already suggested high‐grade pT1 disease. Once complete resection confirmed pTa disease, the patient was appropriately transitioned to intravesical BCG, consistent with standard NMIBC management.

The use of PU in urologic procedures, although less common in modern practice, remains a valuable technique when anatomical challenges arise. It has been reported in patients with urethral strictures, morbid obesity, or penile prostheses, where standard endoscopic approaches are not feasible [2–5]. In our patient′s case, severe scar tissue from burn injuries presented a unique obstacle, which to our knowledge, has not been previously documented as an indication for temporary PU. The ability to bypass the urethra and achieve direct bladder access allowed for the resection of multiple large bladder tumors without the need for open surgery, reducing the risk of tumor dissemination and iatrogenic complications.

Less invasive alternatives, such as optical internal urethrotomy (OTIS) or laser urethrotomy, were considered but were not applicable in this case. These techniques are effective for intrinsic urethral strictures, in which scar tissue narrows the urethral lumen itself. On cystoscopic evaluation, however, the anterior urethra was normal and no true intraluminal stricture was ever identified; the barrier to access was extrinsic, arising from severe tethering and immobility of the overlying skin and subcutaneous tissue due to childhood burn scarring, which prevented the rigid scope from being maneuvered into the posterior urethra despite a patent lumen. Because the limitation was positional and related to soft tissue mobility rather than a true urethral stricture, urethrotomy would not have addressed the underlying access problem, and PU was selected as the most direct solution. Physicians should also recognize the prominent role of en bloc resection of bladder tumors (ERBT) in achieving high‐quality pathologic specimens and complete tumor removal, offering improved specimen integrity, more accurate pathologic staging through preserved detrusor muscle architecture, and a theoretical reduction in tumor cell dissemination compared with conventional piecemeal resection [6, 7]. In appropriately selected patients with solitary, technically accessible tumors, ERBT is an attractive option. In our patient, however, three separate frondular tumors were identified at the left posterior bladder wall, left dome, and right bladder neck. This multifocal distribution across noncontiguous sites, combined with the constrained working environment created by the PU approach, made single‐specimen en bloc excision of each lesion impractical; conventional fractionated resection was therefore performed to ensure complete tumor clearance within a reasonable operative time. Future cases with more limited, unifocal disease amenable to PU‐assisted access may be reasonable candidates for an en bloc technique, which merits consideration where anatomically feasible.

The complications associated with PU are generally minor, as demonstrated by our patient′s superficial wound dehiscence, which was resolved with a return to the operating room for closure. No long‐term complications, such as strictures or fistulae, have been observed. Literature suggests that temporary PU is associated with lower rates of urethral strictures compared with transurethral procedures when rigid scopes must traverse compromised anatomy [1, 3]. Additionally, preservation of the longitudinal blood supply in the bulbar urethra during formation of PU may further reduce the risk of stenosis in select patients [8].

## 4. Conclusion

Temporary PU for bladder tumor resection in patients with complex anatomy, such as severe scarring, is a viable and effective option that can enable complete tumor resection without the limitations of traditional transurethral surgery.

## Funding

No funding was received for this manuscript.

## Consent

Written informed consent was obtained from the patient for the publication of this case report, including relevant clinical details and any accompanying figures. The patient has been assured that all efforts have been made to protect their privacy, and no identifiable information has been disclosed.

## Conflicts of Interest

The authors declare no conflicts of interest.

## Data Availability

Data sharing is not applicable to this article as no datasets were generated or analyzed during the current study.
